# News and Notes

**Published:** 1901-03

**Authors:** 


### *NEWS AND NOTES.*

Dr. W. Monroe Smith is spending some weeks at the Chicago Policlinic.

Dr. Wm. Perrin Nicolson, Dr. V. O. Hardon and Dr. G. H. Noble attended the Pan-American Medical Congress.

Dr. James H. Kellogg, Battle Creek, Mich., was stabbed, but not seriously wounded, by an insane patient December 22.

Dr. Edwin Klebs, for the last four years professor at Rush, Chicago, has settled at Munich since his return to Germany.

A new medical journal has been issued at Havana known as the Revista de la Association Medico Farmac. de la Isla de Cuba.

The Cross of the French Legion of Honor has been presented to Dr. Velde, the German physician at Peking during the siege of the legations.

A fire took place in the library and laboratory of Dr. Howard A. Kelly on December 16, which damaged books and furniture to the extent of $600.

A monument is to be erected by subscription to the eminent French surgeon, L. Ollier, whose death in his seventy-sixth year occurred a short time ago.

Dr. Knapp's Gift.—Dr. Herman Knapp has presented to the New York Ophthalmic and Aural Institute, as a gift, the buildings now occupied by that institution.

AT a meeting of the Société de Chirurgie of Paris, M. Chaput related a case in which a woman had a tampon removed from the abdomen that had been there for seven years.

---

THE authorities at Dawson, Yukon, have telegraphed to the outside for 10,000 more vaccine points for use in enforcing the compulsory vaccination ordinance recently passed.

---

DR. CHAPOT PREVOST, who performed the operation on the exiphopagus monster, has been awarded a sum amounting to about $4,000 as a prize by the Brazilian government.

---

THE osteopath at Beaver Falls, Pa., "Dr." E. E. Pierce, charged with practising medicine and surgery without a license, was found guilty and recommended by the jury to the mercy of the court.

---

PLAGUE IN ENGLAND.—Another death from the plague has occurred among the members of the crew of the British steamer "Friary," which left Alexandria December 22, bound for Hull, by way of Algiers.

---

THE faculty of medicine of the University of Edinburgh, according to the *British Medical Journal*, has formally expressed its disapproval of the publication of laudatory certificates from its graduates, in trade advertisements.

---

DR. W. W. KEEN has been re-elected president of the College of Physicians and Surgeons of Philadelphia. Dr. H. C. Wood has been elected vice-president, Dr. Thomas R. Neilson, secretary, and Dr. Richard H. Harte, treasurer.

---

A "DIPLOMATE" of the notorious Independent Medical College was arrested at Grand Rapids, Mich., December 17, on the charge of practicing medicine without having legally registered. It is likely that this will be made a test case.

---

THE CHICAGO POLICLINIC AND HOSPITAL.—Our subscribers are requested to see the advertisement of the above school on page xxx. Those desiring to take postgraduate course would do well to write to Dr. M. L. Harris, the Secretary.

---

DR. T. D. CROTHERS.—Dr. Thomas D. Crothers, of Hartford, Conn., has been appointed Professor of Diseases of the Brain and Nervous System, with especial reference to the effects of alcoholic and drug poisons, at the New York School of Clinical Medicine.

---

DR. MARY KAVENY, Denver, who recently brought suit against Dr. Clayton Parkhill for $10,000 damages, because of an alleged shortening of one-quarter of an inch in a leg treated by him, has refused to allow X-ray photographs to be taken of the leg, and in consequence the suit will probably be dropped.

---

THE Iowa state board of medical examiners met at Des Moines, December 18, and refused to issue certificate to an osteopath, a graduate of the Still College of Osteopathy, Des Moines. The board has also declined to grant licenses to practice to graduates of schools of the same character at Quincy, Ill., and Kirksville, Mo.

---

DR. CARLOS FINLAY was recently tendered a banquet by the physicians of Havana in token of congratulation that his theory of the transmissibility of yellow fever by the mosquito, which he has been proclaiming for many years in the face of opposition and ridicule, has at last received recognition and acceptance.

---

DR. EDUARDO WILDE, the Argentine Minister, one of the most distinguished representatives from South America, now in Washington, is also a physician. He has been chosen a delegate to the Pan-American Congress which will begin its session in Havana, Cuba, February 4, 1901. Dr. Wilde intends to present a new plan for establishing sanitary regulations in the ports of the whole of America.

---

PROFESSOR CELLI estimates in his new work, "La Malaria," an annual mortality in Italy of 15,000 due to malaria, and states that 5,000,000 acres of good soil remain uncultivated. As a member of the Italian parliament, he proposes to make it obligatory on land-owners and employers to provide every possible agent against the scourge. It is likewise proposed that the government supply sufferers with pure quinine at a trifle above cost price.

---

THE state board of health, Indiana, has considered three points of medical legislation which it is proposed to lay before the general assembly at its coming session: (1) Examination of all applicants for license to practice medicine; (2) that county clerks shall keep on file as a matter of record in their offices the certificates which the state medical board sends to them on which they issue license to medical practitioners; and (3) the enactment of a law defining the phrase "practice of medicine."

---

PROF. JOS. MCFARLAND, the well-known bacteriologist and pathologist of Philadelphia, will hereafter devote his energies to research work in the biological laboratories of Parke, Davis & Co., Detroit. Dr. McFarland is a recognized teacher, writer, and authority on bacteriology, and his ripe experience will render the work he now takes up still more beneficial to the medical profession. We congratulate this enterprising house in having obtained the services of such an accomplished investigator.

PLAGUE IN SAN FRANCISCO.—Prof. Frederick G. Novy, of the University of Michigan, left last week on an important mission for the United States government. He goes in all possible haste to California to investigate the likelihood that the bubonic plague, or "black death," is about to break out there, and, if such be the case, to institute every repressive measure known to medical science. It is understood that Dr. Novy is to carry on the work suddenly interrupted by Dr. Kinyoun's illness.—*Medical News.*

---

AMERICAN MEDICAL MISSIONARY SCHOOL.—Backed by the Battle Creek (Michigan) Sanitarium, the American Medical Missionary College in this city will erect two four-story buildings for its own use in the neighborhood of the Cook County Hospital. These buildings will house the college, hospital, and afford dormitories and dining-halls for the students. The outlay contemplated is $150,000. The college has been in existence five years and was incorporated under the laws of Illinois. Dr. J. H. Kellogg is the chief physician and instructor at the college.—*Medical News.*

---

TEA AND ALCOHOL IN RUSSIA.—The New York Sun, in a recent issue, states that tea was first imported into Russia in 1838. Nearly one pound per year is now consumed on an average by each inhabitant. The total consumption of tea in Russia is 106,000,000 pounds, and the total cost thereof about $88,000,000. For brandy, beer and wine, $550,000,000 are expended. It seems that the use of tea is increasing rapidly relative to the alcoholic beverages. It is worthy of mention that this has taken place since the introduction of machine-made teas from Ceylon and India.

---

NEW MEDICAL SOCIETIES.—Wm. Warren Potter expresses his opinion of these in an editorial in the January *Buffalo Medical Journal:* "One of the great evils of the period is, in our view, the tendency to multiply medical societies. If the medical societies will amalgamate, consolidate or dissolve, so as to reduce their number, they will surely in this way expand and increase their influence. The man who proposes the organization of a new medical society is not only asking for something that is wholly unnecessary, but he is very near being a menace to the best interests of the guild of medicine."

---

JOURNAL OF HYGIENE.—The first number of this new journal, to be devoted to original work in hygiene, has just appeared. The senior editor, Dr. George F. Nuttall, is well known for his excellent work. The papers in this number are varied and valuable. Anopheles and the Distribution of Malaria, Pathogenic Organisms in Milk, Industrial Lead-Poisoning, Carbonic Acid in the Air, Artificial Modification of Toxins, Isolation Hospitals and Scarlet Fever. These, with other short notes, show the wide interests that the new journal will subserve. It is to be published quarterly at a price of $4.—*Medical News.*

---

SMALLPOX AND VACCINATION.—Recently a mail-carrier was found in the Melrose Lodging House, on upper Third avenue, suffering from smallpox. The health board officials promptly removed the patient and vaccinated every one in the building with the exception of two lodgers, who refused vaccination. About a week afterwards, on January 8, one of these men was attacked by smallpox, and on the following day the other, while of the many others who were equally exposed to the disease not one has yet contracted it. The total deaths from smallpox since the beginning of the present outbreak now number sixteen.—*Boston Medical and Surgical Journal.*

---

DURING the current winter semester the number of women on the register of the Berlin University is 371, of whom 253 are

Germans and 118 foreigners. Of the Germans 111 belong to Berlin. Statistics of age are not given, but there is at least one sweet girl undergraduate of sixty-one summers who is repairing the deficiencies of her early education by studying German philology and history. Last winter the number of female students in the University was 431. The cause of the diminution is said to be that stricter regulations have been passed for the admission of Russian women. Women were first admitted to the University in the summer of 1896.

---

THERE is trouble in San Francisco because the Pacific Coast Regular College of Medicine, a night medical school, incorporated in May, 1900, proposes to graduate seven students this month. It is claimed that two of the seven would-be graduates have no certificates showing that they have taken the requisite courses of study and passed the required examination. There is nothing in the law of California to prevent any enterprising person from incorporating himself and a few of his friends as a medical college and thereafter issuing degrees after compliance by the graduates with such requirements as may seem to suit him best. Dr. Dudley Tait, of the State Board of Examiners, says he will oppose the granting of licenses to the graduates of this school.—*Journal A. M. A.*

---

THE Colorado State Medical Society offers a prize of twenty-five dollars for the best essay, if deemed worthy of the prize, pointing out the dangers to public health and morals, especially to young persons, from quackery as promulgated by public advertisements.

The competition is open to all. Essays must be typewritten in the English language, and submitted before May 15th, 1901. Each essay must be designated by a motto; and accompanied by a sealed envelope, bearing the same motto, and enclosing the name and address of the author. The essay receiving the prize will become the property of the society for publication. Others will be returned on application. Essays should be sent to the Literature Committee, Room 315 McPhee Building, Denver, Colo.

ACCORDING to the *Journal of the American Medical Association*, an increase of ten per cent. in the drug business for the year just closed was recorded. The trade had its sensational features, and in some respects was materially different from previous years. The war in China produced material advances in all Chinese drugs, but part of them were lost. In quinine there was an advance from 32 to 39 cents, but large importations of bark produced a reaction to the low point, and the close was heavy. Castor oil jumped up 12 cents, and camphor 16 cents. The latter was due to a large consumption and a reduction in supplies. The increased demand for bismuth for army use caused an advance of nearly 50 cents. The most sensational change was in cocaine, due to various influences. Early in the year it sold from $6.20 down to $3.50, but advanced to $6.75. The enlarged consumption of carbolic acid in the African war reduced European supplies, and prices advanced 50 per cent. The year was not without material declines, among the most noticeable being a drop of 50 cents in salol. The drug sundries had a large sale, but they are handled more extensively now by dry-goods houses and department stores than by wholesalers of drugs. The aggregate business of the year was $10,000,000.

---

CAUSE OF THE QUEEN'S DEATH.—The most authentic account of the cause of the queen's death is that derived from the *British Medical Journal*, as follows: "The queen's health had been failing for the past twelve months. The symptoms were mainly of a dyspeptic kind and were accompanied by impaired general nutrition and periods of insomnia and later by occasional slight and transitory attacks of aphasia, the latter suggesting that the cerebral vessels had become damaged. Dyspepsia, which tended to lower her majesty's original robust constitution, was especially marked during her last visit to Balmoral. It was there that the queen first manifested distinct symptoms of brain fatigue and lost notably in weight. These symptoms continued at Windsor, where, in November and December, 1900, slight aphasic symptoms were first observed. They were always of an ephemeral kind and were not attended by any motor paralysis. The queen suffered unusual fatigue on her journey to Osborne on December 18, showing symptoms of nervous agitation and restlessness which lasted for two days. A few days before her final illness, transient but recurring symptoms of apathy and torpor with aphasic indications and increasing feebleness gave great uneasiness to her attending physicians. On January 16 the queen showed for the first time some symptoms of mental confusion. By an effort of the will, however, her majesty would for a time, as it were, command her brain to work, and the visitor of a few minutes would fail to observe signs of cerebral exhaustion. On Thursday of week before last the mental confusion was more marked, with considerable drowsiness, and a slight flattening was observed on the right side of the face. From this time on aphasia and facial paresis, although incomplete, were permanent. On Friday the queen was a little brighter. On Saturday evening there was a relapse and grave symptoms, which, with remissions, continued until the end. It is important to note that, notwithstanding the great bodily weakness and cerebral exhaustion, the heart's action was steadily maintained to the last, the pulse at times evincing increased tension, but always regular and normal. Frequently in the last few hours of life paresis of the pulmonary nerves set in, but the heart beats steadily to the end. Beyond the slight right facial flattening there was never any motor paralysis. Except for the occasional lapses, the mind cannot be said to have been clouded, and within a few minutes of her death the queen recognized several members of her family."—*Medical News*.

---

THE annual meeting of the Association of Medical Officers of the Army and Navy of the Confederacy will be held in Memphis, Tenn., in connection with the annual reunion of the United Confederate Veterans, May 28th-30th prox.

The Committee of Arrangements have sent out the following circular letter:

MEMPHIS, TENN., March 1, 1901.

DEAR DOCTOR:—The Association of Medical Officers of the Army and Navy of the Confederacy will convene in Memphis, Tenn., May 28-30, 1901, during the meeting of the Confederate Reunion. All surgeons, assistant surgeons, acting assistant surgeons, or contract physicians and hospital stewards, in the army and navy of the Confederate States, and all regular physicians who served honorably in any capacity in the Confederate States army and navy, and all regular physicians who are sons of Confederate veterans, are eligible to membership.

You are cordially invited to attend said meeting and contribute reports of important cases coming under your observation, and any reminiscences worthy of preservation connected with your service in the army or navy of the Confederacy.

If you desire to become a member of the Association, and expect to attend the meeting next May, please fill out the enclosed blank and return same to the Secretary at once, in order that your name may appear on the roll

Respectfully,

G. B. MALONE, M.D.,

Chairman,

281 Main St., Memphis, Tenn.

A. L. ELCAN, M.D.,

Secretary,

Southern Express Building,

Memphis, Tenn.

The enclosed blank alluded to contains space for name in full; time and place of enlistment; rank at time of enlistment; rank at close of war; character of service—army or navy; when and where surrendered; present address, and remarks.

Any further information desired will be most cheerfully furnished by Drs. Malone or Elcan, of Memphis, or Dr. Deering J. Roberts, Secretary of the Association, of Nashville, Tenn.

A recent visit to Memphis elicited the fact that nothing will be left undone to provide for the comfort, enjoyment and pleasure of the survivors of the late war between the States. Every man, woman and child in Memphis is fully enthused and thoroughly aroused, with a full determination that the occasion shall be both eventful and momentous to every one who may be so fortunate as to attend.

The doctors of Memphis will see that their end of the line is fully kept up; and with a uniform railroad rate of one cent per mile over all Southern and Southeastern roads the attendance should surely be a feature of the occasion.

